# Patient-centric soulbound NFT framework for electronic health record (EHR)

**DOI:** 10.1186/s44147-023-00205-9

**Published:** 2023-04-28

**Authors:** Namrta Tanwar, Jawahar Thakur

**Affiliations:** Department of Computer Science, Himachal Pradesh University, Shimla-5, India

**Keywords:** Electronic health record, Ethereum, Blockchain, Interoperability, Scalability, Privacy

## Abstract

Interest in leveraging blockchain technology to boost healthcare and e-health solutions has lately increased. Blockchain has proven to have enormous promise in a range of e-health industries because of its decentralized and reliable nature, including the secure exchange of electronic health records (EHRs) and database access management among numerous medical entities. A unique paradigm known as the “patient-centric approach” places the patient at the center of the healthcare system and gives them complete control over who has access to and can share their personal health information. Strong confidentiality and safety requirements are necessary for health information. Additionally, other concerns must be resolved, such as secrecy, interoperability, scalability, cost-effectiveness, and timeliness. This paper offers a patient-centric privacy-preserving framework for an efficient and safe medical record to address these problems. Based on three parameters transaction cost, execution time, and gas cost. Three blockchain platforms are compared by using the smart contract to find out the suitable platform for the implementation of this framework. Blockchain platforms served as a benchmark for the performance assessment of a designed framework. Although blockchain will not fix every issue in healthcare organizations, it will undoubtedly assist in dramatically reducing some of the most critical ones.

## Introduction

Electronic health records (EHR) have replaced our document-based storage system, yet ownership of such sensitive data remains with unidentified parties, leaving it open to security risks. An electronic health record, or EHR, is a digital record of a patient’s medical history, including a diagnosis, medications, follow-up consultations, allergy records, and lab and test results. The patient’s medical file can contain information about his/her family history. All such information must maintain a high level of confidentiality against the outside environment and may only be disclosed to legitimate entities [1]. These issues might be resolved using a ground-breaking technology like blockchain. A structure for storing data that is secure, confidential, and irritable is provided by the Bitcoin blockchain [2]. Various blockchain technologies are being used for business applications. Two of the most popular blockchains are Ethereum and Hyperledger. Ethereum is well-liked because it offers a permissioned network and fast transaction processing. As indicated by numerous initiatives being undertaken in various nations and industries, governments and relevant industrial sectors are becoming intensely interested in digitizing medical systems. To benefit patients and society, HITECH aims to encourage the broad use of electronic medical records (EHRs). The scientific community’s interest in EHR systems has also been driven by their prospective advantages, including public healthcare administration, online accessibility of health, and patient health data sharing. The 2019 report novel coronavirus (2019-nCoV and COVID-2019) outbreak, in which telemonitoring surveillance and other public healthcare technologies are increasingly deployed to limit the situation, also serves as more evidence of the potential of EHRs [3].

### Electronic health record (EHR)

**Fig. 1:**
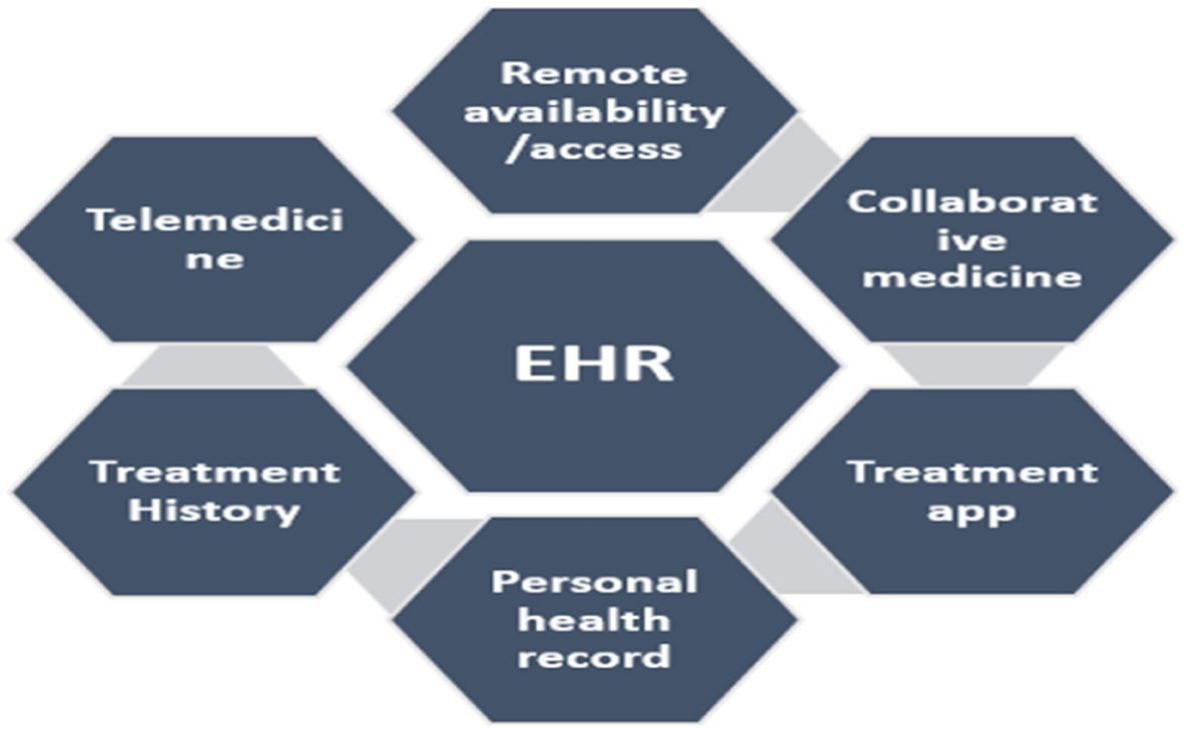
Electronic health record illustration

Electronic health records are digital representations of a patient’s medical history. The digital health record collects, generates, and stores electronic data [4]. EHRs are patient-centered, real-time records that quickly and securely provide access to authorized users to information. The main benefits that innovation is currently offering are to improve customer service, security, and other areas of the medical industry. The programs referred to as digital health record (EHR) and electronic medical record (EMR) systems [5], which record and send massive volumes of medical data every day, provide these advantages. An EHR system is meant to go beyond clinical features and data gathered in a provider’s office and can provide a wider perspective of a patient’s care, even though it incorporates patients’ health and treatment histories. Vital details including a description of each patient’s health, administrative duties, and legal documents could be found in their EHR (Fig. 1).

Even though it contains individual treatment and medical records, an EHR system is intended to extend beyond the conventional clinical information recorded in a company’s office and can encompass a wider view of a child’s treatment. Each patient’s EHR may include important data, such as a summary of their general health, administrative data, and legal papers.

## Blockchain technology

Due to the success of Bitcoin, users can now utilize these technologies in a range of markets and services, such as the financial sector, the Internet of Things, distribution networks, polling, healthcare, and agriculture [6]. Blockchain utilizes a decentralized system that allows for the integration of some fundamental technologies, including distributed consensus, asymmetric cryptography-based digital signatures, and cryptographic hashes [7]. Smart contracts can be used to complete data or transactions without the involvement of any third-party provider trusted authority thanks to the decentralized consensus process of the blockchain.

Blockchain technology is composed of six key elements:

- Decentralized: The fundamental characteristic of blockchain is that global data may be recorded, stored, and modified without the need for centralized nodes.
- Transparent: Blockchain may be trusted since the data stored by the network is accessible to every node and accessible on the most recent data.
- Open source: The majority of blockchain systems are public, the records can be reviewed by anybody, and anyone can utilize blockchain technology to create any applications they like.
- Autonomy: The goal is to transfer from a single individual to the entire system, and nobody can participate in it, thanks to the consensus mechanism that allows each node on the Bitcoin network to transfer or modify data safely.
- Immutable: Any recordings will be kept permanently and cannot be altered until more than 51% of a node is taken over at once.
- Anonymity: With the use of blockchain tech, the issue of node trust has been resolved, allowing for anonymized data movement and even transactional activity [6].

## Blockchain layers

Layer 0, layer 1, layer 2, and layer 3 are the four blockchain layers that make up the blockchain layered structure. The numerous scalable solutions provided to a public blockchain are also described by the 4 blockchain tiers. Scalability refers to a Bitcoin’s ability to handle a high volume of activities concerning the amount of data traffic [8]*.*

### Layer 1

- The majority of functions that maintain a Bitcoin network’s core functionality, including dispute settlement, consensus, computer languages, regulations, and limitations, are carried out by layer 1. It represents the real blockchain.
- Scalability issues usually arise because of the enormous number of tasks that this layer must manage. The amount of computer resources needed to solve and add transactions to a blockchain increases as more users join, leading to higher fees and extended processing times.
- The concern about scalability is somewhat allayed by enhanced consensus approaches like proof-of-stake and the introduction of pooling (the division of computing operations into smaller parts). History has nonetheless demonstrated that they fall short. Layer 1 examples include Ether, Bitcoin, and Solana.

### Layer 2

- It takes more processing power to increase the blockchain’s productivity. However, this calls for the addition of additional nodes, which congest the network. Adding nodes is necessary to maintain a blockchain’s decentralized nature; however, adjusting scalability, decentralization, or bandwidth will have an impact on the other layer 1 factor.
- Consequently, layer 1 cannot be made larger without moving all operations to layer 2, which was added on top of layer 1. By enabling the integration of third-party services with layer 1, this is made possible.
- Layer 1 is updated by a second network, layer 2, which also controls all financial verifications. In the blockchain ecosystem, layer 2 sits atop layer 1 and communicates with it frequently. On the other hand, layer 1 is simply in charge of overseeing the creation and adding of larger blocks to the network.
- Take the distributed system as an illustration of a layer 2 cryptocurrency that has been implemented on the Bitcoin protocol.

## Soulbound token (SBT)

So, because we need to stop the trading of the documents, therefore, here, SBT is used. Soulbound tokens cannot be bought or given to the other person; once you have one, it will remain linked to your private wallet and identity. They are therefore perfect for digitally representing things like credentials, reputation, and records of medical care that cannot be purchased. The token’s owner can then decide who has rights to the information it contains and can also cancel that access as necessary. The ability to manage personal information in the blockchain-enabled form rather than having it stored in a central database makes SBTs an option for people who want the most access to their information [10]. ERC-721 [9], in contrast, proposes a non-fungible token specification that is distinct from exchangeable tokens. This kind of token is distinct from others and may be identified. Particularly, the coupling of contract address and uint256 token ID is hard to crack because every NFT has this variable.

## Related work

Shahnaz [2] carried out a study on cryptocurrency technology and electronic health records. The study was conducted on EHR systems that have issues with data management, security, and authenticity. The author talked about how cryptographic protocols can be applied to change EHR systems and offer a potential remedy for these problems. The study focused on their suggested framework, which would employ blockchain-based to deploy EHR, and would also define granular access controls for users and provide storage facilities for electronic medical records. With the help of the framework, the EHR system now has access to scalable, safe, and essential blockchain-based solutions [2].

As a result, the researcher was able to present a taxonomy for blockchains, describe common blockchain consensus methodologies, review blockchain networks, talk about technical difficulties and recent developments in solving those difficulties, and also identify the recommendations for the future in distributed ledger technology [7].

Wang et al. give an overview, assessment, opportunities, and challenges of the non-fungible token (NFT). The idea behind NFT was inspired by an Ethereum token standard that sought to identify each token by a distinctive symbol. The NFT ecosystems are examined in this study from many angles. We begin by providing a summary of cutting-edge NFT systems before describing their technical elements, protocols, regulations, and required proprieties. After that, we provide a security development and talked about opportunities, difficulties, and design model viewpoints. As far as we are aware, this is the initial comprehensive analysis of the current NFT communities [11].

Gangwal et al. carried out research on layer 2 blockchain protocols. In essence, it is an examination of blockchain layers. We methodically develop a comprehensive taxonomy of these applications and protocols. We go into detail about each layer 2 communication class and explain their approaches, key characteristics, needs, etc. In addition, we describe the problems with various procedures and compare them [12].

Toqeer Ali Syed et al. compare the architecture of blockchains and their applications, highlighting issues and offering suggestions. This research study also offered the full blockchain ecosystem of all the publications that we evaluated and annotated. Various blockchain systems, their agreement models, and their implementations are also analyzed. Finally, we go through important factors that are necessary for blockchain technology to be widely adopted in these important fields in the future [13].

Kuo et al. provide a comparison of distributed ledger platforms so that scientists and software developers working in clinical or universal healthcare information technologies can become familiar with the key technical characteristics of various distributed ledger platforms before designing and implementing cryptocurrency healthcare information applications [14].

## Methods

In this study, both descriptive and qualitative research is done to get an understanding of the topic. A type of research called descriptive research concentrates on describing demographic traits. It collects data that is used to address a range of what, when, and how inquiries. Qualitative research is a method of naturalistic inquiry that also seeks a thorough understanding of social processes. It examines the “why” of numerous human events instead of concentrating on the “what,” and includes case studies, historical research, ground theories, and biographical material. The present state of the art can be determined through study, while analytical research assists in discovering the pertinent facts and information required to fulfill this action. The ultimate objectives of the project require empirical investigation.

### Implementation of proposed framework: SoulBound NFT Smart Contract framework

**Fig. 2:**
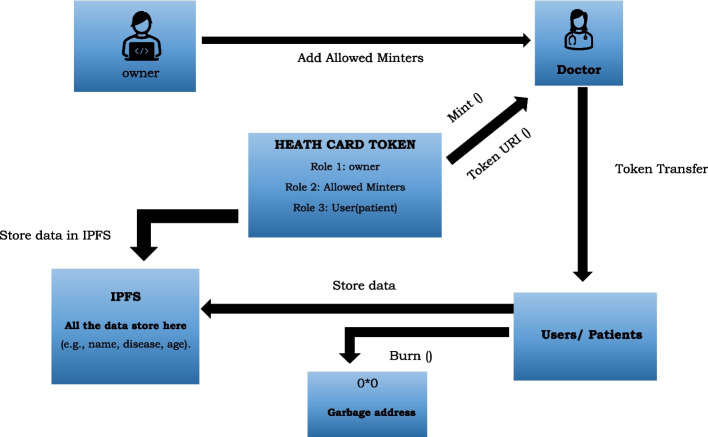
Representation of the proposed framework

The proposed system is built on a blockchain foundation with patients, physicians, and owners all treated as internal entities (Fig. 2).

### Working on the proposed framework

#### Health card token

An HCT is a medium of exchange that the patient will possess; tokens serve as proof of ownership. Since ERC721 provides a standard for NFT, it is employed in this health card token. This sort of token has a unique value and may have a slight difference from another symbol belonging to the same shared ledger.

This health card token consists of three roles: (a) owner, (b) doctor, and (c) patient.

#### Owner

The owner is the administrator who builds the NFT or smart contract; the admin can add the allowed minters but cannot mint the transaction.

#### Minters (doctors)

Authorized doctors can add the patients. The doctor can mint the transaction and can burn the tokens. Also, doctors have access to the patient record 24 × 7.

#### Users/patients

Doctors mint the NFT to the patient. After that, the access is owned by the patient. Patients can validate and view their records.

#### Interplanetary file system (IPFS)

IPFS is a protocol that uses a peer-to-peer network for data storage. It provides secure data storage as data stored on IPFS is protected from any alteration. It uses a cryptographic identifier that protects the data from alteration as any attempt to make the change to the data stored on IPFS could only be done by changing the identifier. The IPFS protocol works in the following way [2]:

- Files stored on IPFS are assigned a unique cryptographic hash.
- Duplicate files are not allowed to exist on the IPFS network.
- A node on the network stores the content and index information of the node.

#### Garbage address (0 × 0)

By calling the burn function to convert the NFT to trash value, the token is rendered unusable and cannot be used again. Because Bitcoin data is unchangeable, if a patient died and had a token or was otherwise present in the blockchain, this garbage address would be assigned to that NFT. In that instance, it would be good to safeguard that NFT or information oxo address. The tokens are no longer of use after the burn function has been applied.

#### Non-fungible tokens

In recent years, the non-fungible tokens (NFT) industry has exploded. The idea behind NFT was inspired by an Ethereum token standard that sought to identify each token by a distinctive symbol. These tokens’ distinctive identifiers can be connected to virtual or digital properties. A sort of cryptocurrency called a non-fungible token (NFT) is derived from Ethereum’s payment systems. NFT was initially put forth in EIP-721 for Ethereum and further improved in EIP-1155.

ERC 721 coin is the NFT token utilized in this contract. NFTs are connected to contracts since they mint based on contracts, and NFTs are found in the user’s or patient’s purse. The ERC721 is made with the aid of the Openzepplin library.

Table 1 lists all the constraints that are implemented in the code for valid and invalid transactions. In the remix, a warning along with “transaction failure” is presented in the output terminal if any constraint is violated.

**Table 1:** Constraints of transaction validation

Valid constraints		
Accounts	Right to do	Enable to do
Owner	• Contract deployment • Doctor approved • Assign a doctor to a patient—grant access	• Does not have minters rights • Patient not registered • Assignee is not a valid doctor • The doctor is not assigned to this patient
Doctors	• Doctor registered successfully • Add patients • Doctors can access records	• Already existing doctor • Does not have the right to add doctors
Patient	• Patient registered successfully • The patient has the right to burn token • The patient can view records and validate them	• Already existing patient • Does not have owner and doctor’s rights

### Contribution of purposed framework

#### Decentralization

The blockchain has made records available in a decentralized format. Each node has a duplicate of the information (only in the view form). In an emergency, doctors can easily obtain the information. Additionally, medical professionals will have access to the patient’s brief history.

#### Patient-centric approach

The NFT will make the patient the owner of their information. Their data cannot be altered without their approval. The patient can verify the information.

#### Global and quick access

Now, for any company, each patient will be assigned a single account or ID with all their past data available. As a result, there will not be any inconsistencies.

#### Interplanetary file system (IPFS)

IPFS is used to store the data, ensuring that it is organized and safe. Inconsistency will be avoided, and information will be accurate and useful.

#### Interoperability

Interoperability is the capacity of different systems to share information or patient data. Due to the number of EHR systems being utilized in so many different institutions, there is no universally accepted standard for them [2]. The intended framework offers a platform where all organizations may access a blockchain-based shared platform, and since every node has a backup of the information, they can access it without any problems.

#### Privacy and security

Privacy is automatically increased thanks to blockchain features like transparency, immutability, decentralization, NFTs, and smart contracts when compared to conventional EHR systems.

#### Smart contract

**Fig. 3:**
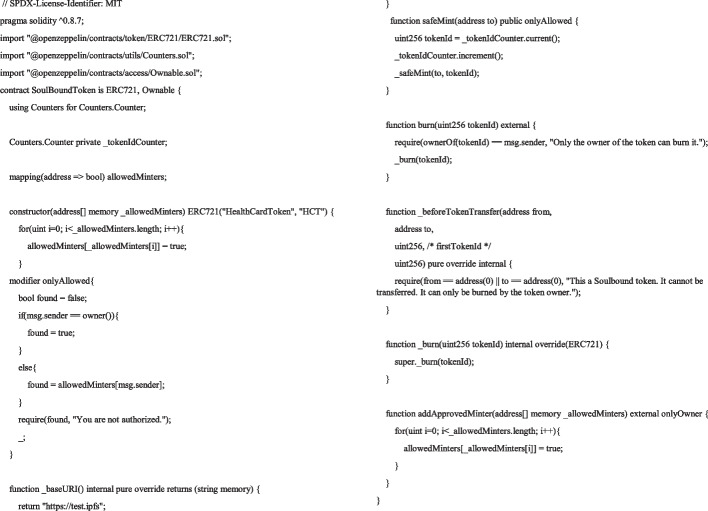
Chain code

A smart contract is a self-executing computer program that can be performed automatically if certain criteria are met. The name of the smart contract (Fig. 3) is “health card token”. Openzepplin library is used to build the ERC721 NFT contract. The modifier is used to validate the transaction means either the transaction is sent by the contract owner or by allowed minters. The Safemint function is used for authorization.

## Results and discussion

Three different networks are utilized to develop the framework and evaluate its interoperability with the primary networks. A group of nodes used to evaluate the Ethereum technology is known as a testnet. On the testnets, tests are carried out to make sure the protocol is operating as intended. Because they are meant to test the standard in a regulated setting, testnets are like mocks. Building tests and delivering them on the testnet are far less expensive than creating decentralized applications and publishing them on the mainnet. This is because we need to pay gas fees (which are actual financial costs) before we can install our contracts on the mainnet. Naturally, when testing your decentralized applications, you do not wish to use real money. Testnets can help in this situation. For blockchain 1-layer networks, Goreli testnet is used and for the 2-layer network, two testnets Binance and Polygon testnets are used (Table 2).

**Table 2:** Archived results

Method	Networks	Average gas cost	Transaction fee	Processing time
NFT smart contract	Ethereum Mainnet	0.00179429	2,836,686	3.7 s
NFT smart contract	Polygon network	0.000000016	96,664	2. 15 s
NFT smart contract	Binance smart chain	0.000000018	21,000	3 s

**Fig. 4:**
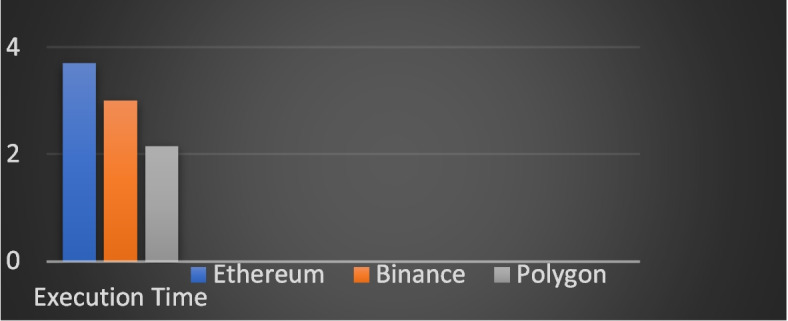
Chart for processing time

By contrasting these test networks, the study concludes that polygon is the most effective platform for installation as it is the fastest of both the bunch and is built on a 2-layer blockchain, which has the key benefit of being cheap and scalable. Additionally, the execution time only takes 2.15 s (Fig. 4), and the typical gas cost is very affordable. Below is the feature comparison table of layer 1 and layer 2 platforms (Table 3).

**Table 3:** Platform features comparison

Features	Ethereum	Polygon network	Binance network
Released	July 30, 2015	June 1, 2020	September 2020
Transaction per second	30	45 tsp	55–60 tsp
Scalability	Low scalability	Highly scalable	Highly scalable
Average gas price	22 Gwei	1 Gwei	5 Gwei
Speed	12 s	Under a second	3–5 s
Transaction fee	Varies	Varies	$0.29–0.50
Consensus	Proof of work (PoW)	Proof of stake (PoS)	PoSA (authority)
Native token	ETH	Matic token	BNB
Other tokens	ERC20	ERC20, ERC721	Fungible and non-fungible tokens

## Conclusions

The present study has concluded that the purpose of the EHR in the health sector is to make the user or patient’s life easier by resolving or providing answers to the problems encountered in the EHR. Patient data can be securely protected by utilizing blockchain technologies. To maintain security and anonymity, blockchain is offering security measures. This framework enables secure, decentralized storage and sharing of medical records. This framework also provides a patient-centric approach and overcomes the interoperability and scalability issues. In the future, a physician validation capability could be developed. Additionally, the patient must give their consent before the organization can share their record with customers. We hope that this review will help to provide more insight into the development and implementation of next-generation EHR systems that will benefit our society.

## Data Availability

The article contains all of the data that were generated or evaluated during this work.

## Abbreviations

EHR: Electronic health record
IPFS: Interplanetary file system
NFTs: Non-fungible tokens
HCT: Health card token
